# Fragile X and Fatal Rhythms: Electroconvulsive Therapy–Induced Ventricular Tachycardia

**DOI:** 10.1016/j.jaccas.2025.105077

**Published:** 2025-08-14

**Authors:** Muhammad Salman Sabri, Nimra Klair, Joshua Wiener, Maria T. Gamero, Drew Johnson

**Affiliations:** a Department of Internal Medicine, Jefferson Abington Hospital, Abington, Pennsylvania, USA; b Department of Cardiology, Thomas Jefferson University Hospital, Philadelphia, Pennsylvania, USA

**Keywords:** electroconvulsive therapy, fragile X syndrome, ventricular tachycardia

## Abstract

**Background:**

Fragile X syndrome (FXS) is associated with autonomic dysfunction and ion channel abnormalities that increase the risk of arrhythmias. Electroconvulsive therapy (ECT), used to treat catatonia in FXS, can trigger a sympathetic surge, potentially inducing ventricular tachycardia.

**Case Summary:**

A 52-year-old man with FXS and catatonia developed recurrent wide complex tachycardia and cardiac arrest during ECT sessions, despite normal electrolytes, no structural heart disease, and a normal baseline electrocardiogram. The patient had recurrent cardiac arrest despite scheduled oral amiodarone 200 mg 3 times a day and premedication with 30 mg of esmolol. Pretreatment with a left stellate ganglion block and intravenous amiodarone infusion was considered before ECT sessions.

**Discussion:**

FXS may increase susceptibility to arrhythmia owing to elevated sympathetic tone and channelopathies associated with fragile X mental retardation protein. ECT may amplify this risk, requiring proactive strategies.

**Take-Home Messages:**

Patients with FXS may require individualized pre-ECT risk assessment. Antiarrhythmic and sympathetic modulation strategies, including stellate ganglion block, may be essential for arrhythmia prevention in high-risk cases.

## Graphical Abstract

**Figure:**
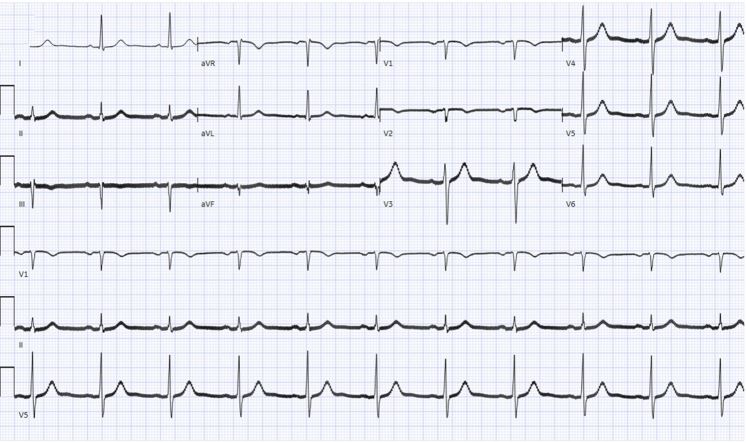

Fragile X syndrome (FXS) is a genetic disorder characterized by a trinucleotide expansion that results in intellectual disability. It is associated with various cardiovascular issues, including hypertension, arrhythmias, and connective tissue disorders such as spontaneous coronary artery dissection and aortic dilatation. Patients with FXS can also develop catatonia due to underlying neurological and psychiatric conditions, which is often managed with electroconvulsive therapy (ECT). While ECT typically causes bradyarrhythmia, evidence suggests excessive sympathetic surge during the procedure can lead to ventricular tachycardia (VT). Reports of VT in patients with FXS are rare. We present a case of VT and cardiac arrest in a patient with FXS after ECT, and the therapeutic dilemma in management of VT.

## History of Presenting Illness

**Figure 1:**
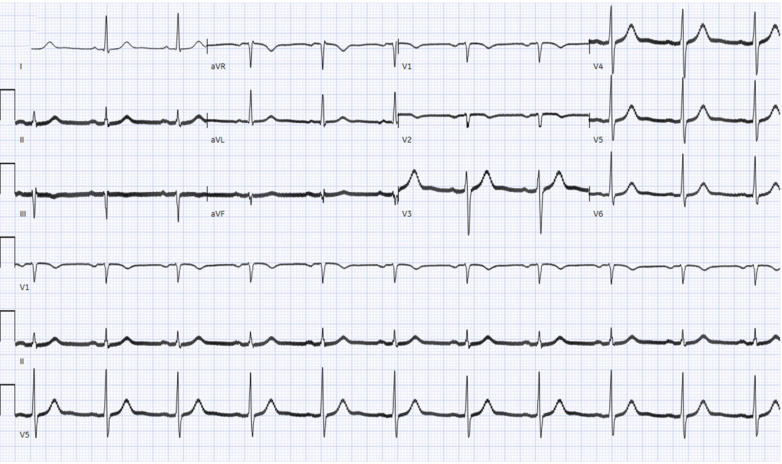
Baseline Electrocardiogram Demonstrating Normal Sinus Rhythm The calculated QT was 421 ms.

**Figure 2:**
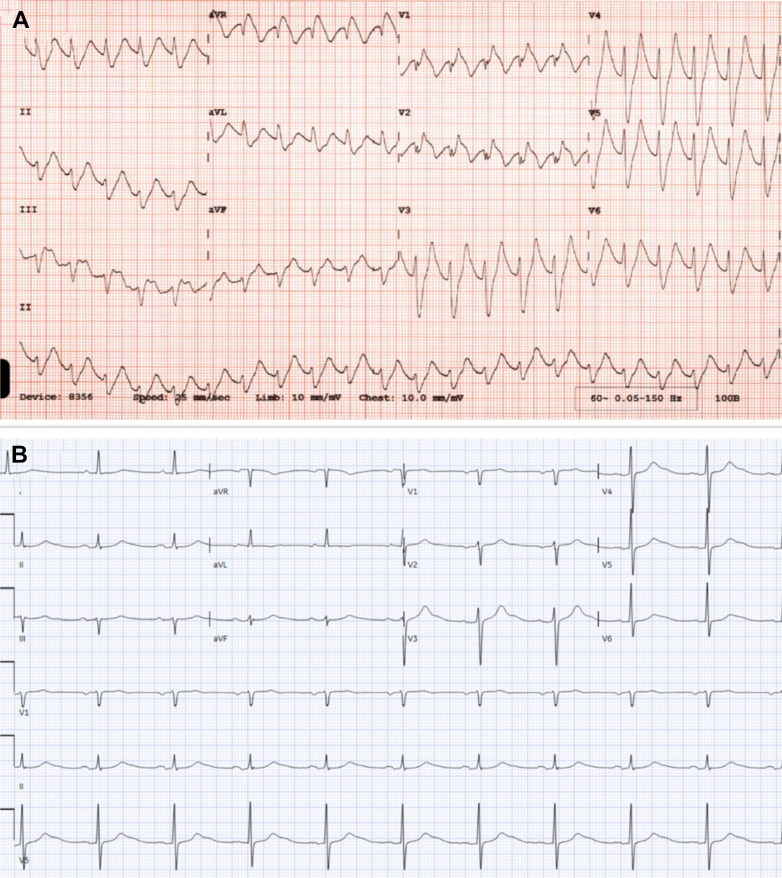
Electrocardiogram Showing Wide-Complex Tachycardia Electrocardiograms demonstrating (A) a wide complex tachycardia and (B) after cardiac arrest with normal sinus rhythm and a calculated QT interval of 440 ms.

A 52-year-old man was admitted for evaluation and management of encephalopathy and catatonia. Because of ongoing catatonia that did not respond to increasing doses of benzodiazepines, ECT was initiated. Before the initiation of ECT, his baseline electrocardiogram (Figure 1) revealed normal sinus rhythm with a QTc interval of 421 ms. Laboratory findings showed normal electrolyte and hemoglobin levels. He was not taking any medications known to prolong the QT interval. During the third ECT session, the patient experienced a pulseless cardiac arrest associated with wide complex tachycardia (Figure 2A), likely VT, necessitating defibrillation and cardiopulmonary resuscitation. Return of spontaneous circulation was achieved within 4 minutes. The patient was intubated during the event.

On physical examination after the arrest, the patient remained intubated and sedated. Cardiovascular examination revealed a regular heart rate and rhythm without murmurs, gallops, or rubs. Lungs were clear to auscultation, the abdomen was soft, and the skin was warm and dry.

## Past Medical History

The patient's history was notable for FXS, hypertension, type 2 diabetes mellitus (not requiring long-term insulin therapy), coronary artery calcifications, and a seizure disorder.

## Investigations

**Figure 3:**
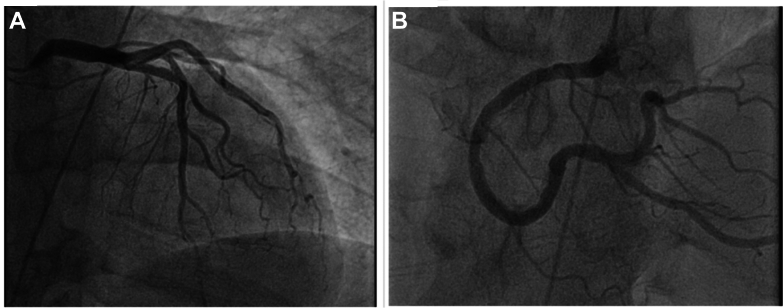
Cardiac Catheterization Cardiac catheterization using the right anterior oblique caudal view showing (A) a patent left anterior descending artery and left circumflex artery as well as (B) a patent right coronary artery.

The electrocardiogram after cardiac arrest (Figure 2B) demonstrated normal sinus rhythm with normal QTc and poor R-wave progression. A transthoracic echocardiogram (subcostal view) (Video 1) showed preserved left ventricular ejection fraction (65%) with small pericardial effusion. Coronary angiography (Figure 3) demonstrated nonobstructive coronary artery disease, including minimal luminal irregularities in the left main and left circumflex arteries, 30% stenosis in the proximal left anterior descending artery, and diffuse mild atherosclerosis in the right coronary artery.

Despite these cardiac events, and after a family discussion owing to the severity of his catatonia, another ECT session was carried out. During that session, the patient experienced a brief episode of VT that resolved spontaneously.

## Management

Given concerns about a proarrhythmic state potentially linked to FXS and the arrhythmogenic nature of ECT, the patient was started on intravenous amiodarone with bolus of 150 mg, later transitioned to oral amiodarone at 200 mg daily. He was also receiving esmolol 30 mg before the ECT sessions. Additionally, aspirin and statin therapy were initiated for management of nonobstructive coronary artery disease.

**Figure 4:**
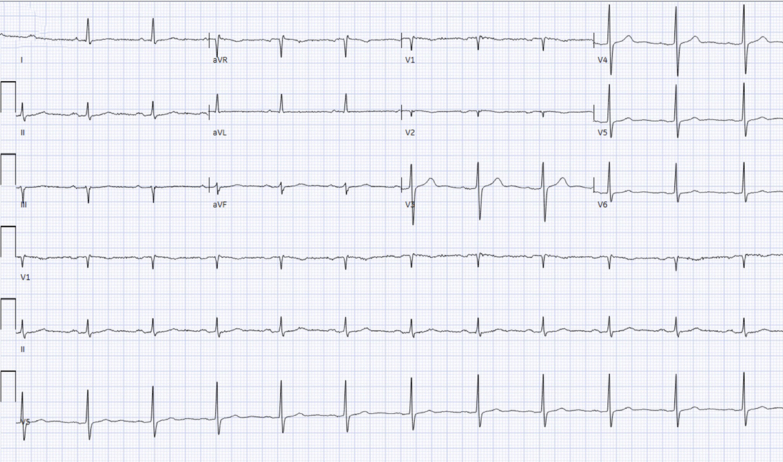
Electrocardiograms Demonstrating Normal Sinus Rhythm and Corrected QT of 410 ms

Despite these measures, the patient suffered a third episode of sustained VT arrest during an ECT session while on amiodarone. Return of spontaneous circulation was achieved after 30 minutes. Electrocardiogram (Figure 4) performed earlier that morning showed normal sinus rhythm with a QTc of 410 ms. The electrophysiology team recommended considering intravenous amiodarone infusion (0.5 mg/min for 24 hours) and performing a stellate ganglion block before further ECT treatments. After this, the patient successfully completed 25 ECT sessions without further major cardiac complications.

## Discussion

FXS is characterized by a deficiency of the fragile X mental retardation protein (FMRP), which plays a critical role in cellular function, including the regulation of ion channels. The absence or reduction of FMRP has been implicated in connective tissue abnormalities, such as valvular heart disease, particularly mitral valve prolapse, as well as vascular fragility that may predispose to arterial dissections.^1^ Additionally, cardiac arrhythmias, including atrial fibrillation and premature ventricular complexes, have been reported in this population.^1^

Recent evidence suggests that individuals with FXS demonstrate elevated baseline heart rates, likely reflecting an imbalance in autonomic tone, as well as increased sympathetic activity and reduced parasympathetic input.^2^ FMRP is also involved in the regulation of multiple ion channels, including sodium, potassium, and calcium channels. Its deficiency may lead to channelopathies, which not only contribute to the neurocognitive phenotype of FXS but may also increase the risk of arrhythmogenesis, including ventricular tachycardia.

ECT is a recognized intervention for severe, treatment-resistant psychiatric conditions and is designed to induce a controlled generalized cerebral seizure.^3^^,^^4^ The autonomic response to ECT involves an initial parasympathetic surge, often resulting in transient bradyarrhythmias, followed by a sympathetic surge during seizure induction, which may lead to tachyarrhythmias.^3^ Although ECT-induced VT has been previously reported (Table 1), these cases invariably involve additional risk factors such as electrolyte abnormalities, structural cardiac disease, or use of agents such as atropine that are known to predispose to arrhythmias.^3^ To date, no documented cases have described ECT-induced VT occurring in the absence of these contributing factors.

**Table 1:** Summary of Case Reports on ECT-Related Ventricular Tachycardia

First Author	Comorbidities	Arrhythmia	Proposed Mechanism	Postulated Prevention
Larsen et al^6^	AF and CHF	NSVT for 17 s after ECT	Sympathetic surge and atropine use	Avoid atropine
Urabe et al^7^	Schizophrenia	VT for 30 s after ECT in setting of suxamethonium anesthesia	Suxamethonium-induced hyperkalemia	Use of thiamylal 250 mg and vecuronium 8 mg instead of suxamethonium along with magnesium prophylaxis prevented future episodes
Kim et al^8^	Unknown	NSVT of unknown duration	Catecholamine-induced	Resolved with IV landiolol; no recurrence
Bailey et al^9^	Catatonia	VT for 9 min	Hyperkalemia from succinylcholine	Avoiding succinylcholine before ECT
Usui et al^10^	Depression	VT for unknown duration	Adrenaline surge	Prophylaxis with esmolol
Koga et al^11^	Drug-resistant depression	VT for 10 s	Adrenaline surge causing tachycardia and hypertension	No future ECT sessions
Pal et al^12^	Psychosis	VT for 2 min	Hypokalemia and adrenaline surge	Managing hypokalemia and hypomagnesemia
Grover and Aggarwal^13^	Schizophrenia	Recurrent VT	Atropine and adrenaline surge	Avoid atropine before ECT
Su et al^5^	HFrEF, AF, TAVR	NSVT that converted to AF	Adrenaline surge	Treatment with esmolol/amiodarone
Sabri et al (our case)	FXS, HTN, DM2, CAD, seizure disorder	Multiple NSVTs and sustained pulseless VTs of variable duration	FMRP-related channelopathy and sympathetic surge	Addition of standing oral amiodarone, premedication with esmolol and consideration of IV amiodarone infusion 24 h before ECT and stellate ganglion block

AF = atrial fibrillation; CAD = coronary artery disease; CHF = congestive heart failure; ECT = electroconvulsive therapy; FMRP = fragile X mental retardation protein; HFrEF = heart failure with reduced ejection fraction; HTN = hypertension; DM2 = type 2 diabetes mellitus; IV = intravenous; NSVT = nonsustained ventricular tachycardia; TAVR: transcatheter aortic valve replacement; VT = ventricular tachycardia.

In the present case, our patient with FXS developed VT in the setting of ECT despite having normal electrolyte levels before and after arrhythmia, no structural heart disease on echocardiogram or catheterization, a normal baseline QTc, and no exposure to proarrhythmic medications such as atropine. We postulate that the underlying autonomic dysregulation in FXS marked by elevated baseline sympathetic tone and potential FMRP-related channelopathy synergistically increased the susceptibility to ECT-induced VT in our patient. The additional sympathetic surge induced by the seizure likely exceeded this patient's arrhythmic threshold, resulting in life-threatening VT.

Despite the occurrence of cardiac arrest, ECT sessions continued given the presence of profound catatonia, severe functional impairment, and alignment with family wishes. Initial management with standard antiarrhythmic therapy and beta-blocker prophylaxis was insufficient to prevent recurrence. However, a strategy involving pretreatment with intravenous amiodarone 24 hours before ECT and stellate ganglion block was successful in preventing further episodes of ECT-induced VT.

Although there is limited literature on the use of ECT in patients with FXS,^5^ this case highlights the potential risk of fatal arrhythmias in this unique population. The findings underscore the importance of recognizing FXS as a condition that may inherently predispose individuals to arrhythmias due to elevated sympathetic tone and possible ion channel dysfunction. Future research is warranted to better understand the arrhythmogenic risk in FXS, to guide the development of prophylactic strategies for VT in the setting of ECT, and to explore sympathetic modulation techniques such as stellate ganglion blockade as potentially more effective interventions than antiarrhythmics alone, particularly in cases not associated with scar-mediated VT.

## Conclusions

This case highlights FXS as a potentially underrecognized risk factor for VT, particularly in the context of ECT. The interplay of autonomic dysregulation increased sympathetic tone, and potential FMRP-related ion channel abnormalities may elevate arrhythmogenic risk in this population. Given this vulnerability, thorough pre-ECT cardiac evaluation including baseline electrocardiogram and assessment for structural or conduction abnormalities should be considered. Continuous telemetry monitoring during and after ECT sessions is essential for early detection and management of arrhythmias. This case also underscores the urgent need for further research to better understand the electrophysiological phenotype of FXS and to develop evidence-based guidelines for arrhythmia prevention and monitoring in this high-risk group undergoing ECT.

**Take-Home Messages**

• FXS may predispose patients to VT owing to underlying autonomic dysfunction and ion channel abnormalities (channelopathies), even in the absence of structural heart disease.
• ECT-induced sympathetic surge can trigger VT in vulnerable populations such as patients with FXS, warranting careful preprocedural risk assessment.
• Sympathetic modulation strategies, including stellate ganglion block, may be essential adjuncts to antiarrhythmic therapy in preventing ECT-induced VT in high-risk cases.

## Funding Support and Author Disclosures

The authors have reported that they have no relationships relevant to the contents of this paper to disclose.

## Appendix

Video 1 Transthoracic Echocardiogram (Subcostal View) Shows Normal Left Ventricular Systolic and Diastolic Function, With No Evidence of Valvular Disease or Chamber Dilation A small pericardial effusion is present, without signs of cardiac tamponade.

## References

[1] Tassanakijpanich N., Cohen J., Cohen R., Srivatsa U.N., Hagerman R.J. (2020). Cardiovascular problems in the fragile X premutation. Front Genet.

[2] Heilman K.J., Harden E.R., Zageris D.M., Berry-Kravis E., Porges S.W. (2011). Autonomic regulation in fragile X syndrome. Dev Psychobiol.

[3] Deng P.Y., Klyachko V.A. (2021). Channelopathies in fragile X syndrome. Nat Rev Neurosci.

[4] Baroud E., Bond J.B., Luccarelli J., Olusunmade M., Henry M.E., Abrams A.N. (2022). Safe administration of electroconvulsive therapy in a patient with catatonia and neuropsychiatric lupus comorbid with fragile X syndrome. J ECT.

[5] Su S., Shah P., Jassal D.S. (2023). Ventricular tachycardia triggered by electroconvulsive therapy: case report and review of the literature. Clin Case Rep.

[6] Larsen J.R., Hein L., Strömgren L.S. (1998). Ventricular tachycardia with ECT. J ECT.

[7] Urabe K., Koguchi T., Ishikawa K. (2001). [A case of ventricular tachycardia immediately after electroconvulsive therapy in a schinzophrenic patient] Article in Japanese. Masui.

[8] Kim C., Sakamoto A., Ogawa R. (2005). Effect of landiolol on nonsustained ventricular tachycardia during electroconvulsive therapy. Anesth Analg.

[9] Bailey C., Venn R., Panayiotou S. (2006). Electroconvulsive therapy for catatonia resulting in cardiac arrest. Eur J Anaesthesiol.

[10] Usui C., Hatta K., Yokoyama T. (2008). Possible effect of beta-blocker on the prevention of ventricular tachycardia during electroconvulsive therapy. Psychiatry Clin Neurosci.

[11] Koga Y., Mishima Y., Momozaki M., Hiraki T., Ushijima K. (2011). A case of nonsustained ventricular tachycardia immediately following modified electroconvulsive therapy in a depressive patient. J Anesth.

[12] Pal A., Samanta S., Samanta S., Wig J. (2015). Sustained ventricular tachycardia after electroconvulsive therapy: can it be prevented?. Indian J Psychol Med.

[13] Grover S., Aggarwal S. (2020). Recurrent ventricular tachycardia during the electroconvulsive therapy procedure: a case report. Indian J Psychiatry.

